# Novel *HLA* allele associations with susceptibility, staging, symptomatic state, autoimmune hepatitis and hepatocellular carcinoma events for primary biliary cholangitis in the Japanese population

**DOI:** 10.3389/fimmu.2023.1151502

**Published:** 2023-05-31

**Authors:** Seik-Soon Khor, Kazuko Ueno, Nao Nishida, Minae Kawashima, Yosuke Kawai, Yoshihiro Aiba, Yuki Hitomi, Masao Nagasaki, Minoru Nakamura, Katsushi Tokunaga

**Affiliations:** ^1^Genome Medical Science Project, National Center for Global Health and Medicine, Tokyo, Japan; ^2^The Research Center for Hepatitis and Immunology, Research Institute, National Center for Global Health and Medicine, Ichikawa, Japan; ^3^Database Center for Life Science (DBCLS), Research Organization of Information and Systems, Chiba, Japan; ^4^Clinical Research Center, National Hospital Organization (NHO) Nagasaki Medical Center, Omura, Japan; ^5^Department of Human Genetics, Research Institute, National Center for Global Health and Medicine, Tokyo, Japan; ^6^Medical Research Center for High Depth Omics, Medical Institute of Bioregulation, Kyushu University, Fukuoka, Japan; ^7^Center for Genomic Medicine, Graduate School of Medicine, Kyoto University, Kyoto, Japan; ^8^Department of Hepatology, Nagasaki University Graduate School of Biomedical Sciences, Omura, Japan; ^9^Headquarters of Primary Biliary Cholangitis (PBC) Research in NHO Study Group for Liver Disease in Japan (NHOSLJ), Clinical Research Center, NHO Nagasaki Medical Center, Omura, Japan

**Keywords:** primary biliary cholangitis, *HLA*, autoimmune hepatitis, hepatocellular carcinoma, Scheuer staging system

## Abstract

Primary biliary cholangitis (PBC) is a rare autoimmune disease with a clear predisposition for human leukocyte antigen *(HLA)-DR/DQ*-associated loss of immune tolerance for the E2 component of the pyruvate dehydrogenase complex. Three-field-resolution *HLA* imputation of 1,670 Japanese PBC patients and 2,328 healthy controls was conducted using Japanese population-specific *HLA* reference panels. Eighteen previously reported Japanese PBC-associated *HLA* alleles were confirmed and extended to 3-field-resolution, including HLA-DRB1*08:03 to HLA-DRB1*08:03:02, HLA-DQB1*03:01 to HLA-DQB1*03:01:01, HLA-DQB1*04:01 to HLA-DQB1*04:01:01 and HLA-DQB1*06:04 to HLA-DQB1*06:04:01. In addition, additional significant novel *HLA* alleles were identified, including 3 novel susceptible *HLA-DQA1* alleles: HLA-DQA1*03:03:01, HLA-DQA1*04:01:01, HLA-DQA1*01:04:01 and 1 novel protective *HLA-DQA1* allele, HLA-DQA1*05:05:01. In addition, PBC patients carrying HLA-DRB1*15:01:01 and HLA-DQA1*03:03:01 would have a higher predisposition toward developing concomitant autoimmune hepatitis (AIH). Further, late-stage and symptomatic PBC shared the same susceptible *HLA* alleles of HLA-A*26:01:01, HLA-DRB1*09:01:02 and HLA-DQB1*03:03:02. Lastly, HLA-DPB1*05:01:01 was identified as a potential risk *HLA* allele for development of hepatocellular carcinoma (HCC) in PBC patients. In conclusion, we have extended the current knowledge of *HLA* allele associations to 3-field resolution and identified novel *HLA* allele associations with predisposition risk, staging, symptomatic state, and AIH and HCC events for Japanese PBC patients.

## Introduction

Primary biliary cholangitis (PBC), previously known as primary biliary cirrhosis, is a complex autoimmune disease that predominantly affects middle-aged women, with a sex ratio of 9 females to 1 male (1). Clinical manifestations of PBC include chronic and progressive destruction of the small bile duct, granulomatous lymphocytic cholangitis with seroreactivity for antimitochondrial antibodies (AMA) against the E2 component of the pyruvate dehydrogenase complex (2). Common symptoms of PBC include, but are not limited to, fatigue (80% of cases) (3), pruritus (20–70% of cases) (4) and jaundice (25% of end-stage cases)(5). Other complications of PBC (6) include portal hypertension (ascites, varices, hepatic encephalopathy), fat malabsorption, fat deposits and osteoporosis/osteomalacia.

The etiopathogenesis of PBC indicates a genetically predisposed autoimmune disease with the possibility of involving still-unknown xenobiotics or infections (7). An international genome-wide meta-analysis (8, 9) of 5 European and 2 East-Asian cohorts (PBC Consortia, Canadian PBC Consortium, Chinese PBC Consortium, Italian PBC Study Group, Japan PBC-GWAS Consortium, US PBC Consortium, and UK-PBC Consortium) reported human leukocyte antigen (HLA) genes as the strongest associated genes. *HLA*-targeted gene approach studies have reported the strongest susceptible HLA allele associations for *HLA* class II genes, with HLA-DRB1*08:03 and HLA-DQB1*06:01 in Japanese PBC (10–12) and HLA-DRB1*08:01 in Italian PBC (13), US PBC (14) and UK PBC (15).

Autoimmune hepatitis (AIH) is a disease of the hepatic parenchyma (16) that sometimes arises in patients with PBC. According to the Japan AIH National Survey(17), the prevalence of AIH is 23.9 per 100,000 individuals. HLA-DRB1*04:01 has been reported as the *HLA* allele most closely associated with both European (17) and Japanese (18) AIH, while HLA-DRB1*04:05 associations have been reported for Japanese (19), Korean (20) and Latin-American (21) AIH.

Hepatocellular carcinoma (HCC) is the most common form of liver cancer, with hepatitis B virus (HBV) and hepatitis C virus (HCV) infections as two of the most prominent risk factors for HCC development (22). Studies have suggested that cirrhotic PBC represents a rare precursor for HCC development (23–25). Among cases of late-stage PBC, HCC shows an incidence of around 5.9% and occurs predominantly in males (26). A meta-analysis of 8 different studies reported HLA-DRB1*07 and HLA-DRB1*12 associations with the risk of HCC, while HLA-DRB1*07, HLA-DRB1*12 and HLA-DRB1*15 alleles were associated with significantly increased risks of HCC in Asians (27). HLA-DQB1*03:01 and HLA-DQB1*06:02 are reportedly associated with Taiwanese HCV-HCC (28), while HLA-A*33:03 is reportedly associated with Japanese HBV-HCC (29).

**Figure 1:**
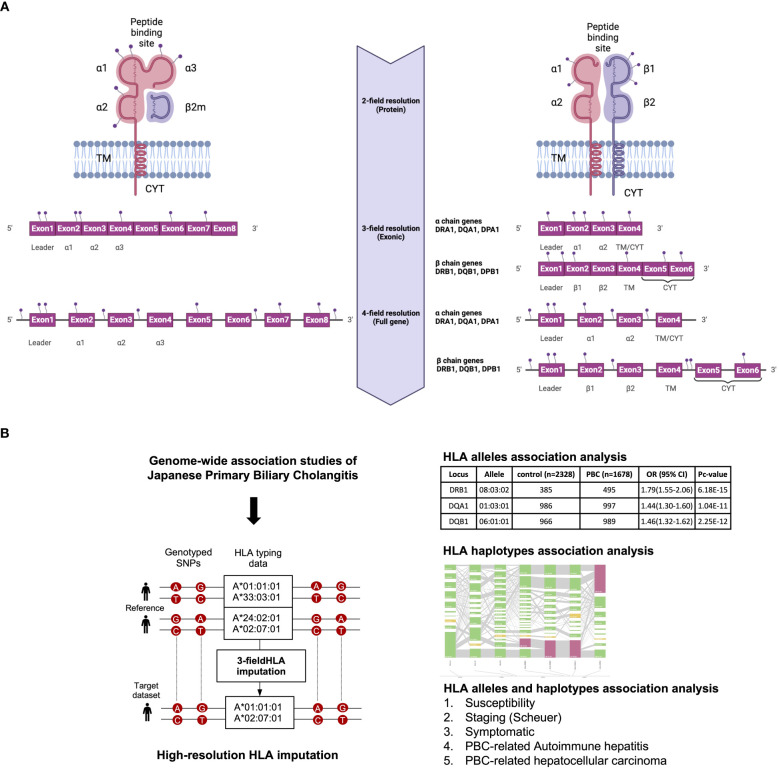
**(A)** Schematic representation of HLA allele resolutions in relation to the mutation sites on the HLA class I and II genes. The 2-field resolution covers the changes in amino acid sequences of the encoded protein for HLA alleles while the 3-field resolution of HLA alleles takes into account changes in all exons that differ by synonymous nucleotide substitutions. Finally, the 4-field resolution encompasses differences in sequence polymorphism in the 5’ or 3’ untranslated regions and introns. α1/2/3, alpha chain; β1, beta chain; β2m, beta2 microglobulin; TM, transmembrane region; CYT, cytoplasmic tail. **(B)** Schematic representation of the study design. 3-field HLA imputation were performed using the SNP genotyping data from GWAS of Japanese PBC. HLA alleles association test were carried out by stratifying based on susceptibility, staging (Scheuer), symptomatic, PBC with AIH and PBC with HCC.

All the above-mentioned *HLA* studies have reported a maximum of 2-field-resolution *HLA* association studies that mainly focused on variations in the core exons of HLA genes, namely exons 2 and 3 for *HLA* class I genes and exon 2 for *HLA* class II genes (**Figure 1**). The present study investigated 3-field-resolution *HLA* allele associations (taking into consideration all variants in the exonic regions of *HLA* class I and class II genes) with susceptibility for PBC, staging of PBC, symptomatic state of PBC, PBC-associated AIH (PBC-AIH) and PBC-associated HCC (PBC with HCC) (**Figure 1**).

## Methods

### Population and SNP genotyping

A total of 1,670 Japanese PBC patients and 2,328 Japanese healthy controls were recruited through the Japan PBC-GWAS Consortium, which comprises 31 hospitals participating in the Japanese National Hospital Organization Study Group for Liver Disease in Japan (NHOSLJ) and 24 university hospitals participating in the gp210 Working Group in Intractable Liver Disease Research Project Team of the Ministry of Health and Welfare in Japan, as described previously (30, 31). The extensive collection of clinical data for Japanese PBC patients and SNP genotyping of Japanese PBC patients and healthy controls are carried out through research undertaken as Japan PBC-GWAS Consortium activities.

Single Nucleotide Polymorphism (SNP) genotyping was performed using the Affymetrix Axiom Japonica v1 (Toshiba, Tokyo, Japan) (32) and Affymetrix Axiom Genome-Wide ASI 1 (Affymetrix, Santa Clara, California, USA) genotyping arrays, as described in previous publications (31, 33). SNP-level and individual-level quality controls were provided using PLINK v1.90 (www.cog-genomics.org/plink/1.9/) (34) with individual call rate ≥98%, SNP call rate ≥99%, minor allele frequency ≥5%, and consistency with Hardy–Weinberg equilibrium (P ≥1.0E-6). Duplicate or closely related samples were removed when identity by descent for each sample pair (pi-hat > 0.1).

### Scoring of chronic hepatitis

Of the total 1,670 Japanese PBC patients, liver biopsies were performed in 1,115 patients and histologically evaluated based on the Scheuer’s classification (35): stage 1, florid duct lesion; stage 2, destruction of limiting plate with piecemeal necrosis or ductular proliferation; stage 3, presence of fibrous septa which is called scarring; stage 4, cirrhosis (**Table 1**).

**Table 1:** Clinical characteristics of Japanese PBC cases.

	PBC (n=1670)
**Age (years), median(range)**	57 (22-89)
**Female (%)**	85.9
**AST (IU/L), (mean ± SD)**	55 ± 88
**ALP (IU/L), (mean ± SD)**	580 ± 411
**IgM (mg/dL), (mean ± SD)**	353 ± 257
**IgG (mg/dL), (mean ± SD)**	1640 ± 519
**Anti-gp210 antibodies positive (%)**	25.9
**Anti-mitochondrial antibodies positive (%)**	88.3
**Anti-nuclear antibodies positive (%)**	72.5
**Scheuer stage 1-2/3-4 (%)**	81.8/18.2
Severity	
**aPBC/s0,1, 2 (%)**	66.4/33.6
Concomitant disease	
**autoimmune hepatitis (%)**	7.2
**other autoimmune diseases (%)**	20.9
Clinical outcome	
**hepatocellular carcinoma (%)**	3.1
**liver transplantation and/or death by hepatic failure (%)**	8.4

### Symptom classification of PBC

Of the total of 1,670 Japanese PBC patients, 1,337 were further classified into the different stages of severity based on clinical symptoms (**Table 1**): asymptomatic PBC (aPBC) = PBC without pruritus, without varices, without ascites, without encephalopathy, and without jaundice; s0 = positive symptoms of portal hypertension, such as esophageal varices; s1 = positive symptoms of pruritus; and s2 = positive symptoms of jaundice (T.bil. > 2.5 mg/dl).

### Clinical diagnosis of PBC-AIH and PBC with HCC

The diagnosis of PBC-AIH is based on the criteria defined by Poupon et al., (36, 37): i) ALT >5 times the upper limit of normal (ULN); ii) IgG >1.5 times ULN; iii) The positive anti-smooth muscle antibody (ASMA), iv) liver biopsy specimen showing moderate to severe periportal or periseptal lymphocytic piecemeal necrosis The presence of HCC was evaluated by ultrasound sonography (UST), computed tomography (CT) or magnetic Resonance Imaging (MRI).

### Measurement of autoantibody testing

Anti-mitochondrial and anti-gp210 antibodies were measured by ELISA, while anti-nuclear antibodies were measured by immunofluorescence test (IF), as described elsewhere (38). 1:100 diluted sera were used for ELISA and 1/40 to 5120 diluted sera were used for IF.

### HLA genotyping and HLA imputation

The 3-field-resolution *HLA* imputation reference consisting of 418 Japanese healthy individuals was generated using the HIBAG R package (v3.16; Xiuwen Zheng) (39, 40) using the SNP genotyping data Affymetrix Axiom Japonica v1 (Toshiba) (32) and Affymetrix Axiom Genome-Wide ASI 1 (Affymetrix) genotyping arrays and high-resolution HLA genotyping data by AllType assay (One Lambda, West Hills, CA, USA). The AllType assay (One Lambda) was designed to cover the full length of five HLA genes (*HLA-A*, *-C*, *-B*, *-DPA1*, *-DQA1*). High-resolution *HLA* genotyping was carried out following vendor instructions for *HLA* gene-specific amplification, *HLA* library preparation, *HLA* template preparation, *HLA* template purification and loading of the *HLA* template onto an ion 530v1 chip (Thermo Fisher Scientific, Waltham, MA, USA) in the ion chef (Thermo Fisher Scientific) followed by final sequencing on the ion S5 machine (Thermo Fisher Scientific). *HLA* genotype assignments were carried out using HLATypeStream Visual (TSV v2.0; One Lambda) and NGSengine^®^ (v2.18.0.17625; GenDX, Utrecht, the Netherlands). Demultiplexing of barcodes and base-calling was carried out in Torrent Suite version 5.8.0 (Thermo Fisher Scientific). Raw fastq reads were extracted using the FileExporter function in Torrent Suite version 5.8.0. *HLA* genotype assignments were carried out using two different types of software, namely HLATypeStream Visual (TSV v2.0; One Lambda) as the default software for the Alltype™ NGS Assay and NGSengine^®^ (v2.18.0.17625; GenDX). The default analysis parameters and healthy metrics threshold was applied for TSV2.0 while we applied the “ignore regions” function in NGSengine to eliminate known sequencing error sites such as homopolymer sites in the ion S5 system. Three- and 4-field *HLA* allele genotyping was determined by comparing reads with IPD-IMGT/*HLA* database release 3.40.0 (https://www.ebi.ac.uk/ipd/imgt/hla/).

SNP data from 3,998 samples were extracted from an extended MHC (xMHC) region ranging from 25,759,242 to 33,534,827 bp based on the hg19 position. Three-field *HLA* imputation was carried out using the HIBAG R package (39) and our in-house 3-field Japanese HLA imputation reference (40). Post-imputation quality control of call threshold (call threshold > 0.5) was applied to remove low-quality samples. In total, we successfully imputed the number of unique *HLA* alleles as follows: *HLA-A*, n=14; *HLA-C*, n=19; *HLA-B*, n=33; *HLA-DRB1*, n=29; *HLA-DQB1*, n=14; *HLA-DQA1*, n=18; *HLA-DPA1*, n=7; and *HLA-DPB1*, n=14.

### Statistical analysis

Case-control and clinical phenotype *HLA* allele association tests, *HLA* haplotype estimations, case-control *HLA* haplotype association tests and Hardy–Weinberg equilibrium tests were prepared and analyzed using the Bridging ImmunoGenomics Data Analysis Workflow Gaps (BIGDAWG) R package (v3.0.3; Derek Pappas, Steve Mack, Jill Hollenbach)(41). The default parameters of BIGDAWG were used except for the manual specification of *HLA* haplotypes for testing. Rare *HLA* alleles with an expected count < 5 were combined into a common class.

## Results

### Clinical characteristics of the 1,670 Japanese PBC cases

A total of 1,670 Japanese PBC patients with extensive clinical information and 2,328 healthy controls were recruited through Japan PBC-GWAS Consortium (**Table 1**). To identify *HLA* alleles potentially associated with the severity of PBC (please refer to the Methods for classification definitions), patients showing Scheuer 0, 1 and 2 were grouped as the early-stage PBC group, while patients showing Scheuer 3 and 4 were grouped as the late-stage PBC group, resulting in 808 of 982 patients (82.3%) showing early-stage PBC and 174 of 982 patients (17.5%) showing late-stage PBC. Similarly, *HLA* profiles were compared between 892 of 1,337 (66.7%) asymptomatic PBC patients and 445 of 1,337 (33.3%) symptomatic PBC patients (please refer to the Methods for classification definitions). In addition, 116 of 1,670 (7.0%) PBC-AIH cases and 49 of 1,670 (2.9%) PBC with HCC cases were compared with their respective negative group.

### Associations of *HLA* alleles and *HLA* haplotypes with Japanese PBC cases

**Figure 2:**
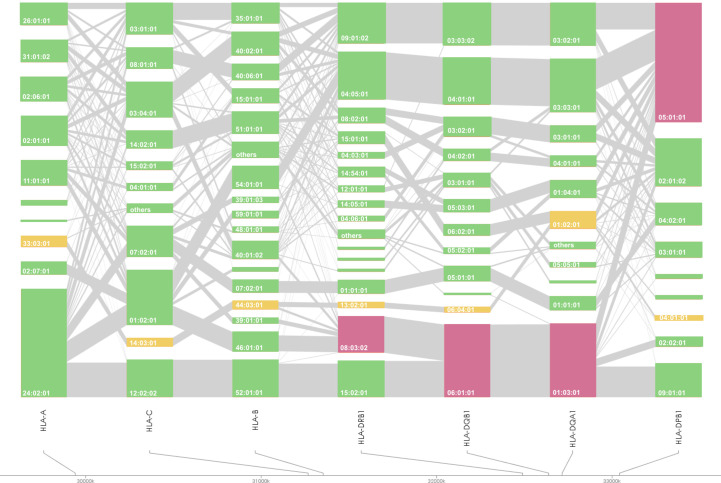
*HLA* allele linkage distributions of *HLA-A, -C, -B, -DRB1, -DQA1, -DQB1*, and *-DPB1* in Japanese PBC. The heights of the bars show the number of samples carrying the particular *HLA* alleles, and grey lines connecting orange bars indicate the proportion of linkage between different orange bars. Susceptible and protective *HLA* haplotypes are highlighted in magenta and yellow, respectively.

*HLA* association studies for the 8 *HLA* genes were carried out comparing *HLA* allele frequencies in the 1,670 Japanese PBC patients and 2,328 healthy controls (**Table 2**). A total of 1 *HLA-A*, 2 *HLA-C*, 5 *HLA-B*, 10 *HLA-DRB1*, 8 *HLA-DQA1*, 7 *HLA-DQB1*, 1 *HLA-DPA1* and 3 *HLA-DPB1* alleles were significantly associated with Japanese PBC after correction for multiple testing. The most significant susceptible *HLA* alleles were HLA-DRB1*08:03:02, HLA-DQB1*06:01:01, HLA-DQA1*01:03:01 and HLA-DPB1*05:01:01 (**Table 2**). *HLA* haplotype analysis confirmed strong linkage among the above-mentioned *HLA* alleles, with the strongest *HLA* haplotype associations (**Table 3**; **Figure 2**) for HLA-DRB1*08:03:02-HLA-DQA1*01:03:01-HLA-DQB1*06:01:01 but weaker *HLA* haplotype association (**Table 3**; **Figure 2**) after adding *HLA-DPB1* allele with HLA-DRB1*08:03:02-HLA-DQA1*01:03:01-HLA-DQB1*06:01:01-HLA-DPB1*05:01:01. In contrast, the most significant protective *HLA* alleles (**Table 2**; **Figure 2**) were HLA-DRB1*13:02:01, HLA-DQB1*06:04:01, HLA-DQA1*01:02:01 and HLA-DPB1*04:01:01. All these protective *HLA* alleles formed strong *HLA* haplotypes (**Table 3**; **Figure 2**) with HLA-DRB1-13:02:01-HLA-DQA1*01:02:01-HLA-DQB1*06:04:01, showing a weaker association after including HLA-DPB1*04:01:01 with HLA-DRB1*13:02:01-HLA-DQA1*01:02:01-HLA-DQB1*06:04:01-HLA-DPB1*04:01:01 (**Table 3**).

**Table 2:** Association analysis of *HLA-A, -C, -B, -DRB1, -DQA1, -DQB1, -DPA1* and *-DPB1* in Japanese PBC patients and Japanese healthy controls.

Locus	Allele	control (2n=4656)	%	PBC (2n=3306)	%	OR (95%CI)	p.value	Pc-value
A	33:03:01	427	9.2	139	4.2	0.43 (0.35–0.53)	2.22E-16	2.66E-15
C	01:02:01	849	18.2	695	21.0	1.19 (1.07–1.34)	1.93E-03	3.29E-02
C	14:03:01	419	9.0	106	3.2	0.33 (0.27–0.42)	2.22E-16	3.77E-15
B	07:02:01	324	7.0	165	5.0	0.70 (0.58–0.86)	3.30E-04	9.24E-03
B	15:01:01	412	8.8	185	5.6	0.61 (0.51–0.73)	6.08E-08	1.70E-06
B	40:02:01	308	6.6	301	9.1	1.42 (1.20–1.68)	3.56E-05	9.96E-04
B	44:03:01	418	9.0	106	3.2	0.34 (0.27–0.42)	2.22E-16	6.22E-15
B	46:01:01	258	5.5	246	7.5	1.37 (1.14–1.65)	5.72E-04	1.60E-02
DRB1	01:01:01	336	7.2	175	5.3	0.72 (0.59–0.87)	6.51E-04	1.56E-02
DRB1	04:05:01	619	13.3	596	18.1	1.44 (1.27–1.63)	4.69E-09	1.13E-07
DRB1	04:06:01	170	3.7	68	2.1	0.56 (0.41–0.74)	4.32E-05	1.04E-03
DRB1	08:02:01	190	4.1	192	5.8	1.45 (1.18–1.80)	3.31E-04	7.94E-03
DRB1	08:03:02	385	8.3	456	13.8	1.78 (1.54–2.06)	1.71E-15	4.11E-14
DRB1	11:01:01	110	2.4	36	1.1	0.46 (0.30–0.67)	3.28E-05	7.86E-04
DRB1	12:01:01	189	4.1	88	2.7	0.65 (0.50–0.84)	8.88E-04	2.13E-02
DRB1	13:02:01	395	8.5	80	2.4	0.27 (0.21–0.34)	2.22E-16	5.33E-15
DRB1	14:03:01	68	1.5	14	0.4	0.29 (0.15–0.52)	6.75E-06	1.62E-04
DRB1	15:01:01	352	7.6	152	4.6	0.59 (0.48–0.72)	1.10E-07	2.64E-06
DQA1	01:01:01	335	7.2	176	5.3	0.73 (0.60–0.88)	8.11E-04	1.30E-02
DQA1	01:02:01	736	15.9	221	6.7	0.38 (0.32–0.45)	2.22E-16	3.55E-15
DQA1	01:03:01	986	21.3	924	28.1	1.45 (1.30–1.61)	2.60E-12	4.16E-11
DQA1	01:04:01	233	5.0	224	6.8	1.38 (1.14–1.68)	7.86E-04	1.26E-02
DQA1	03:03:01	714	15.4	673	20.4	1.41 (1.26–1.59)	5.20E-09	8.32E-08
DQA1	04:01:01	114	2.5	139	4.2	1.75 (1.35–2.27)	1.04E-05	1.66E-04
DQA1	05:05:01	172	3.7	62	1.9	0.50 (0.37–0.67)	2.25E-06	3.60E-05
DQB1	03:01:01	450	9.7	182	5.5	0.54 (0.45–0.65)	1.32E-11	1.72E-10
DQB1	04:01:01	618	13.3	597	18.1	1.44 (1.27–1.63)	4.90E-09	6.37E-08
DQB1	04:02:01	141	3.0	154	4.7	1.56 (1.23–1.99)	1.48E-04	1.93E-03
DQB1	05:01:01	358	7.7	196	5.9	0.76 (0.63–0.91)	2.35E-03	3.05E-02
DQB1	06:01:01	966	20.7	916	27.7	1.46 (1.32–1.63)	5.90E-13	7.67E-12
DQB1	06:02:01	342	7.3	142	4.3	0.57 (0.46–0.69)	1.99E-08	2.59E-07
DQB1	06:04:01	388	8.3	75	2.3	0.26 (0.20–0.33)	2.22E-16	2.89E-15
DPA1	02:02:02	1260	37.7	644	42.1	1.20 (1.06–1.36)	3.23E-03	1.61E-02
DPB1	02:01:02	1143	24.5	616	18.6	0.70 (0.63–0.79)	3.94E-10	5.12E-09
DPB1	04:01:01	308	6.6	60	1.8	0.26 (0.19–0.35)	2.22E-16	2.89E-15
DPB1	05:01:01	1697	36.4	1476	44.7	1.41 (1.28–1.54)	1.52E-13	1.97E-12

OR, odds ratio; 95%CI, 95% confidence interval; Pc-value, multiple testing-corrected p-value; binned, rare HLA alleles with expected count < 5, combined into a common class.

**Table 3:** *HLA* haplotype analysis of *HLA-A, -C, -B, -DRB1, -DQA1, -DQB1, -DPA1* and -*DPB1* in Japanese PBC patients and Japanese healthy controls.

A~B	Control	%	PBC	%	OR (95%CI)	P-value	Pc-value
33:03:01~44:03:01	375	8.1	93	2.8	0.33 (0.26–0.42)	2.22E-16	4.88E-15
24:02:01~07:02:01	270	5.8	134	4.1	0.69 (0.55–0.85)	4.70E-04	1.03E-02
11:01:01~15:01:01	130	2.8	55	1.7	0.59 (0.42–0.82)	9.89E-04	2.18E-02
24:02:01~40:02:01	82	1.8	93	2.8	1.61 (1.18–2.21)	1.61E-03	3.54E-02
C~B	Control	%	PBC	%	OR (95%CI)	P-value	Pc-value
14:03:01~44:03:01	418	9.0	106	3.2	0.34 (0.27–0.42)	2.22E-16	3.77E-15
04:01:01~15:01:01	158	3.4	62	1.9	0.54 (0.40–0.74)	4.66E-05	7.92E-04
07:02:01~07:02:01	324	7.0	165	5.0	0.70 (0.58–0.85)	3.14E-04	5.33E-03
01:02:01~46:01:01	254	5.5	246	7.4	1.39 (1.16–1.68)	3.20E-04	5.43E-03
03:04:01~40:02:01	260	5.6	248	7.5	1.37 (1.14–1.65)	5.62E-04	9.55E-03
08:01:01~40:06:01	171	3.7	171	5.2	1.43 (1.14–1.79)	1.14E-03	1.95E-02
A~C~B	Control	%	PBC	%	OR (95%CI)	P-value	Pc-value
33:03:01~14:03:01~44:03:01	375	9.3	93	3.2	0.33 (0.26–0.42)	2.22E-16	4.44E-15
11:01:01~04:01:01~15:01:01	120	3.0	37	1.3	0.43 (0.29–0.63)	4.00E-06	8.00E-05
24:02:01~07:02:01~07:02:01	270	6.7	135	4.7	0.69 (0.56–0.86)	5.97E-04	1.19E-02
DRB1~DQB1	Control	%	PBC	%	OR (95%CI)	P-value	Pc-value
13:02:01~06:04:01	388	8.3	75	2.3	0.26 (0.20–0.33)	2.22E-16	3.55E-15
08:03:02~06:01:01	385	8.3	456	13.8	1.77 (1.53–2.05)	2.73E-15	4.37E-14
04:05:01~04:01:01	617	13.3	596	18.0	1.44 (1.27–1.63)	5.10E-09	8.17E-08
15:01:01~06:02:01	342	7.3	142	4.3	0.57 (0.46–0.69)	1.99E-08	3.19E-07
08:02:01~04:02:01	85	1.8	108	3.3	1.82 (1.35–2.45)	3.78E-05	6.05E-04
04:06:01~03:02:01	170	3.7	68	2.1	0.55 (0.41–0.74)	3.84E-05	6.15E-04
12:01:01~03:01:01	151	3.2	62	1.9	0.57 (0.42–0.77)	1.94E-04	3.10E-03
01:01:01~05:01:01	336	7.2	176	5.3	0.72 (0.60–0.88)	6.92E-04	1.11E-02
DRB1~DQB1~DPB1	Control	%	PBC	%	OR (95%CI)	P-value	Pc-value
13:02:01~06:04:01~04:01:01	268	6.1	40	1.3	0.20 (0.14–0.28)	2.22E-16	4.44E-15
04:05:01~04:01:01~05:01:01	345	7.9	389	12.4	1.67 (1.43–1.95)	3.55E-11	7.10E-10
08:03:02~06:01:01~05:01:01	197	4.5	254	8.1	1.88 (1.55–2.29)	5.17E-11	1.03E-09
15:01:01~06:02:01~02:01:02	144	3.3	40	1.3	0.38 (0.26–0.55)	3.59E-08	7.18E-07
04:06:01~03:02:01~02:01:02	121	2.8	44	1.4	0.51 (0.35–0.72)	9.10E-05	1.82E-03
01:01:01~05:01:01~04:02:01	242	5.5	115	3.7	0.66 (0.52–0.83)	2.60E-04	5.20E-03
08:03:02~06:01:01~02:02:01	79	1.8	95	3.0	1.71 (1.25–2.35)	4.01E-04	8.03E-03
DRB1~DQA1~DQB1	Control	%	PBC	%	OR (95%CI)	P-value	Pc-value
13:02:01~01:02:01~06:04:01	388	8.3	75	2.3	0.26 (0.20–0.33)	2.22E-16	3.33E-15
08:03:02~01:03:01~06:01:01	385	8.3	456	13.8	1.77 (1.53–2.05)	2.73E-15	4.10E-14
04:05:01~03:03:01~04:01:01	617	13.3	595	18.0	1.44 (1.27–1.63)	6.30E-09	9.44E-08
15:01:01~01:02:01~06:02:01	342	7.3	142	4.3	0.57 (0.46–0.69)	1.99E-08	2.99E-07
04:06:01~03:01:01~03:02:01	170	3.7	68	2.1	0.55 (0.41–0.74)	3.84E-05	5.77E-04
08:02:01~04:01:01~04:02:01	85	1.8	107	3.2	1.80 (1.34–2.43)	5.25E-05	7.88E-04
01:01:01~01:01:01~05:01:01	336	7.2	176	5.3	0.72 (0.60–0.88)	6.92E-04	1.04E-02
A~C~B~DRB1~DQA1~DQB1~DPB1	Control	%	PBC	%	OR (95%CI)	P-value	Pc-value
33:03:01~14:03:01~44:03:01~13:02:01~01:02:01~06:04:01~04:01:01	223	4.8	31	0.9	0.19 (0.12–0.28)	2.22E-16	1.78E-15
24:02:01~07:02:01~07:02:01~01:01:01~01:01:01~05:01:01~04:02:01	187	4.0	86	2.6	0.64 (0.49–0.83)	6.28E-04	5.03E-03
24:02:01~12:02:02~52:01:01~15:02:01~01:03:01~06:01:01~09:01:01	382	8.2	341	10.3	1.29 (1.10–1.50)	1.24E-03	9.94E-03

OR, odds ratio; 95%CI, 95% confidence interval; Pc-value, multiple testing-corrected p-value; binned, rare HLA alleles with expected count < 5, combined into a common class.

### Associations of *HLA* alleles and *HLA* haplotypes with Japanese PBC-AIH cases

*HLA* association studies of the 8 *HLA* genes were undertaken to compare *HLA* allele frequencies in the 116 PBC-AIH cases as compared to 1,537 non-PBC-AIH cases (**Table 4**). 7 HLA alleles including HLA-B*59:01:01, HLA-C*15:02:01, HLA-DRB1*04:05:01, HLA-DQB1*04:01:01, HLA-DQB1*06:01:01, HLA-DQA1*01:03:01 and HLA-DQA1*03:03:01 were significantly associated with susceptibility to PBC-AIH after adjusting for multiple corrections. *HLA* haplotype showed strong DR-DQ associations with HLA-DRB1*04:05:01-HLA-DQB1*04:01:01 and HLA-DRB1*04:05:01-HLA-DQA1*03:03:01-HLA-DQB1*04:01:01 (**Table 4**).

**Table 4:** HLA association analysis and haplotype analysis of *HLA-A, -C, -B, -DRB1, -DQA1, -DQB1, -DPA1* and *-DPB1* in Japanese PBC patients with AIH and Japanese PBC patients without AIH.

Locus	Allele	Non-PBC-AIH (2n=3074)	%	PBC-AIH (2n=232)	%	OR (95%CI)	P-value	Pc-value
B	59:01:01	80	2.6	14	6.0	2.40(1.23–4.35)	2.46E-03	3.69E-02
C	15:02:01	92	3.0	15	6.5	2.24(1.18–3.97)	3.95E-03	4.35E-02
DRB1	04:05:01	528	17.2	68	29.6	2.02(1.47–2.74)	2.79E-06	3.62E-05
DQB1	04:01:01	528	17.2	69	29.7	2.04(1.49–2.77)	1.61E-06	1.77E-05
DQB1	06:01:01	873	28.4	43	18.5	0.57(0.40–0.81)	1.21E-03	1.33E-02
DQA1	01:03:01	880	28.8	44	19.0	0.58(0.40–0.82)	1.37E-03	1.23E-02
DQA1	03:03:01	599	19.6	74	31.9	1.92(1.42–2.59)	7.24E-06	6.51E-05
Haplotype	Alleles	Non-PBC-AIH (2n=3074)	%	PBC-AIH (2n=232)	%	OR (95%CI)	P-value	Pc-value
DRB1~DQB1	04:05:01~04:01:01	527	17.1	69	29.7	2.05(1.5–2.77)	1.49E-06	1.78E-05
DRB1~DQA1~DQB1	04:05:01~03:03:01~04:01:01	526	17.1	69	29.7	2.05(1.5–2.78)	1.37E-06	1.51E-05

AIH, autoimmune hepatitis; OR, odds ratio; 95%CI, 95% confidence interval; Pc-value, multiple testing-corrected p-value; NS, not significant; binned, rare HLA alleles with expected count < 5, combined into a common class.

### HLA-A*26:01:01 association with late-stage PBC (Scheuer 3 and 4)

*HLA* association studies of the 8 *HLA* genes were carried out comparing *HLA* allele frequencies in the 808 patients in the early-stage PBC group (Scheuer 1 and 2) with the 174 patients in the late-stage PBC group (Scheuer 3 and 4) (**Supplementary Table 1**). HLA-A*26:01:01 and HLA-B*55:02:01 and HLA-C*03:04:01 were significantly associated with susceptibility to the staging of PBC (**Supplementary Table 1**). *HLA* haplotype analysis of *HLA* class I alleles (**Supplementary Table 2**) revealed significant *HLA* haplotype associations with HLA-A*26:01:01-HLA-B*40:02:01 and HLA-A*26:01:01- HLA-C*03:04:01-HLA-B*40:02:01.

### HLA-DRB1*09:01:02, HLA-DQB1*03:03:02 and HLA-DQA1*03:02:01 associations with symptomatic PBC in the Japanese population

HLA association studies of the 8 *HLA* genes were carried out comparing *HLA* allele frequencies in the 892 asymptomatic PBC patients with the 445 symptomatic PBC patients (s0, s1, s2) (**Supplementary Table 3**). HLA-DRB1*09:01:02, HLA-DQB1*03:03:02 and HLA-DQA1*03:02:01 were significantly associated with susceptibility to symptomatic PBC after adjusting for multiple comparisons (**Supplementary Table 3**). Strong *HLA* haplotype of the significant *HLA* alleles was confirmed (**Supplementary Table 4**) with HLA-DRB1*09:01:02-HLA-DQB1*03:03:02 and HLA-DRB1*09:01:02-HLA-DQA1*03:02:01-HLA-DQB1*03:03:02.

### No *HLA* association with PBC with HCC

**Supplementary Table 5** presents a comparison of demographic and biochemical values between PBC with HCC and those without HCC. Our analysis found no statistically significant differences between the two groups in the variables examined. *HLA* association studies of the 8 *HLA* genes were carried out comparing HLA allele frequencies between the 49 PBC with HCC patients and 1,604 PBC without HCC patients (**Supplementary Table 5**). No *HLA* allele remained significant after adjusting for multiple comparisons (**Supplementary Table 6**). However, when we performed age-matched HLA association testing with 49 PBC with HCC patients and 49 PBC without HCC patients (**Supplementary Table 7**), HLA-DRB1*09:01:02, HLA-DQB1*03:03:02, HLA-DRB1*09:01:02-HLA-DQB1*03:03:02 remained borderline significant after adjusting for multiple comparisons. HLA-DQB1*03:01 and HLA-DQB1*06:02 are reportedly associated with Taiwanese HCV-HCC (28), while HLA-A*33:03 is reportedly associated with Japanese HBV-HCC (29).

## Discussion

Genome-wide association studies (GWAS) of PBC in populations of different backgrounds, such as Japanese (30, 31) and Caucasian (42, 43), have consistently reported that the strongest SNP associations are located in the *HLA* class II region. *HLA* gene-targeted studies have confirmed the strong associations of *HLA* class II alleles such as HLA-DRB1*08:01 for Caucasian PBC(15); HLA-DRB1*08 and HLA-DRB1*02 for Italian PBC(13); HLA-DRB1*08:03, HLA-DQB1*03:01, HLA-DQB1*04:01 and HLA-DQB1*06:04 for Japanese PBC (12); and HLA-DRB1*08:03, HLA-DQB1*03:01 and HLA-DPB1*17:01 for Chinese PBC (44). In this study, we expanded on previous knowledge from 2-field *HLA* allele associations (12) to 3-field *HLA* allele associations incorporating all variants in *HLA* exonic regions. We have extended the previously known *HLA* allele associations (12) in Japanese PBC from HLA-DRB1*08:03 to HLA-DRB1*08:03:02, HLA-DQB1*03:01 to HLA-DQB1*03:01:01, HLA-DQB1*04:01 to HLA-DQB1*04:01:01 and HLA-DQB1*06:04 to HLA-DQB1*06:04:01 (**Table 2**).

In addition, with the increase in detection power after extending the size of Japanese PBC and healthy control samples, besides replicating all 18 significant *HLA* alleles reported by Yasunami et al., (12) we identified an additional significant novel *HLA* alleles after adjusting for multiple corrections. The strongest associated novel *HLA* alleles (**Table 2**) included 3 novel susceptible *HLA-DQA1* alleles, HLA-DQA1*03:03:01, HLA-DQA1*04:01:01, HLA-DQA1*01:04:01, and 1 novel protective *HLA-DQA1* allele, HLA-DQA*05:05:01. In addition, HLA-A*33:03:01 and HLA-B*44:03:01 were identified as 2 novel protective *HLA* class I alleles. Both these protective *HLA* alleles showed strong linkage disequilibrium (LD) with strong *HLA* haplotypes of HLA-A*33:03:01-HLA-B*44:03:01 (**Table 3**). This would represent an extension of associations from *HLA* class II alleles with HLA-A*33:03:01-HLA-C*14:03:01-HLA-B*44:03:01-HLA-DRB1*13:02:01-HLA-DQA1*01:02:01-HLA-DQB1*06:04:01-HLA-DPB1*04:01:01 (**Table 3**).

Previous studies focusing on AIH identified susceptible *HLA* allele associations for HLA-DRB1*04:01 and HLA-DRB1*04:05 in a Japanese population (18), HLA-DRB1*03:01 and HLA-DRB1*04:01 in a European population (45), and HLA-DRB1*04:04, -DRB1*04:05 and -DRB1*13:01 in Latin-American populations (21, 46–48). In a European childhood AIH-1 study (49), HLA-DRB1 alleles carriers are reported to associate with higher onset age, higher IgG levels, median smooth muscle antibodies (SMA) level and anti-nuclear antibodies (ANA). The present study of PBC-AIH confirmed the reported HLA associations of HLA-DRB1*04:01 and HLA-DRB1*04:05 with Japanese AIH (18) (**Table 4**) suggesting that AIH in PBC carries AIH-associated risk HLA alleles.

We have observed similar *HLA* allele associations between late-stage PBC and symptomatic PBC. HLA-A*26:01:01 (**Supplementary Table 1**) was associated with late-stage PBC (Scheuer 3 and 4) and although not significant after adjusting for multiple corrections, the same *HLA* allele is also associated with a higher risk of symptomatic PBC. On the other hand, HLA-DRB1*09:01:02 and HLA-DQB1*03:03:02 (**Supplementary Table 3**), which were associated with a higher risk of developing symptomatic PBC, shared a similar tendency of associations with late-stage PBC with HLA-DRB1*09:01:02 and HLA-DQB1*03:03:02 (**Supplementary Table 1**).

We identified that HLA-DPB1*05:01:01 was associated with PBC with HCC, although this association did not remain significant after adjusting for multiple comparisons (**Supplementary Table 6**). HLA-DPB1*05:01 is reportedly strongly associated with a higher risk of chronic hepatitis B (CHB) in the Japanese population, in which 15–40% of CHB patients eventually develop cirrhosis and/or HCC (50). Although HCC in PBC is rare, studies have suggested that cirrhotic PBC is a rare precursor of HCC development (23–25) and HLA-DPB1*05:01 would represent a shared-risk *HLA* allele for HCC development in both CHB and PBC.

Among the 140 PBC patients who underwent liver transplantation or died of hepatic failure (end-stage PBC), 92.3% were positive for AMA, while the AMA positivity was 88.2% in the group of 1392 PBC patients who did not progress to end-stage PBC, indicating that AMA positivity is not significantly associated with PBC progression to end-stage. HLA association analysis in PBC patients with end-stage and those without progression to end-stage did not reveal any significant HLA allele associations after multiple corrections. Nevertheless, HLA-DPB1*05:01:01 was marginally associated with an increased risk of liver transplantation or death from liver failure (**Supplementary Table 7**).

1- and 2-field *HLA* alleles associations with PBC have been confirmed in multiple population backgrounds. Our results confirmed and extended the current understanding of *HLA* allele associations by investigating 3-field *HLA* alleles associations with predisposition risk, staging, symptomatic status, AIH and HCC events for PBC patients in the Japanese population.

## Data availability statement

The GWAS data summary statistics are deposited in the National Bioscience Database Center (NBDC): https://humandbs.biosciencedbc.jp/hum0261-v2.

## Ethics statement

The studies involving human participants were reviewed and approved by Japanese National Hospital Organization Study Group for Liver Disease in Japan (NHOSLJ). The patients/participants provided their written informed consent to participate in this study.

## Author contributions

S-SK, MiN, KT conceived and planned the experiments. YA and MiN contributed to sample preparation. S-SK, KU, NN, MK, YK, YH and MaN contributed to interpretation of the results. S-SK took the lead in writing the manuscript. All authors contributed to the article and approved the submitted version.

## Supplementary material

Supplementary Table 1 Association analysis of *HLA-A*, *-C*, *-B*, *-DRB1*, *-DQA1*, *-DQB1*, *-DPA1* and *-DPB1* in Japanese PBC patients with Scheuer Stage 0–2 and Japanese PBC patients with Scheuer Stage 3–4. Abbreviations: OR = odd ratio; 95%CI = 95% confidence interval; Pc-value = multiple testing-corrected p-value; binned = rare HLA alleles with expected count < 5, combined into a common class.

Supplementary Table 2 HLA haplotype analysis of *HLA-A*, *-C*, *-B*, *-DRB1*, *-DQA1*, *-DQB1*, *-DPA1* and *-DPB1* in Japanese PBC patients with Scheuer Stage 0–2 and Japanese PBC patients with Scheuer Stage 3–4. Abbreviations: OR = odds ratio; 95%CI = 95% confidence interval; Pc-value = multiple testing-corrected p-value; binned = rare HLA alleles with expected count < 5, combined into a common class.

Supplementary Table 3 Association analysis of *HLA-A*, *-C*, *-B*, *-DRB1*, *-DQA1*, *-DQB1*, *-DPA1* and *-DPB1* in asymptomatic Japanese PBC patients and s0, s1 and s2 Japanese PBC patients. Abbreviations: OR = odds ratio; 95%CI = 95% confidence interval; Pc-value = multiple testing-corrected p-value; binned = rare HLA alleles with expected count < 5, combined into a common class; aPBC = asymptomatic PBC (i.e., without itching, without varices, without ascites, without encephalopathy, without jaundice); s0 = positive symptoms of portal hypertension such as esophageal varices; s1 = positive itching; s2 = positive jaundice (T.bil. > 2.5 mg/dl).

Supplementary Table 4 HLA haplotype analysis of *HLA-DRB1*, *-DQA1* and *-DQB1* in asymptomatic Japanese PBC patients and s0, s1 and s2 Japanese PBC patients. Abbreviations: OR = odds ratio; 95%CI = 95% confidence interval; Pc-value = multiple testing-corrected p-value; binned = rare HLA alleles with expected count < 5, combined into a common class.

Supplementary Table 5 Demographic and biochemical values of PBC with HCC versus PBC without HCC. Abbreviations: ALT: alanine transaminase, ALP: alkaline phosphatase
